# Early Intravenous Beta-Blockade with Esmolol in Adults with Severe Traumatic Brain Injury (EBB-TBI): Protocol for a Phase 2a Intervention Design Study

**DOI:** 10.1007/s12028-023-01755-9

**Published:** 2023-06-12

**Authors:** Matt Thomas, Kati Hayes, Paul White, Aravind Ramesh, Lucy Culliford, Gareth Ackland, Anthony Pickering

**Affiliations:** 1 Intensive Care Unit, North Bristol NHS Trust, Bristol, UK; 2 School of Data Science and Mathematics, University of the West of England, Bristol, UK; 3 GW4 Clinical Academic Training Programme for Health Professionals, Faculty of Health Sciences, University of Bristol, Bristol, UK; 4 Bristol Trials Centre, Bristol Medical School (PHS), University of Bristol, Bristol, UK; 5 Translational Medicine and Therapeutics, William Harvey Research Institute, Queen Mary University of London, London, UK; 6 School of Physiology, Pharmacology and Neuroscience, University of Bristol, Bristol, UK

**Keywords:** Brain injuries (traumatic), Adrenergic beta-antagonists, Intensive care units, Models (statistical)

## Abstract

Traumatic brain injury is a leading cause of death and disability worldwide. Interventions that mitigate secondary brain injury have the potential to improve outcomes for patients and reduce the impact on communities and society. Increased circulating catecholamines are associated with worse outcomes and there are supportive animal data and indications in human studies of benefit from beta-blockade after severe traumatic brain injury. Here, we present the protocol for a dose-finding study using esmolol in adults commenced within 24 h of severe traumatic brain injury. Esmolol has practical advantages and theoretical benefits as a neuroprotective agent in this setting, but these must be balanced against the known risk of secondary injury from hypotension. The aim of this study is to determine a dose schedule for esmolol, using the continual reassessment method, that combines a clinically significant reduction in heart rate as a surrogate for catecholamine drive with maintenance of cerebral perfusion pressure. The maximum tolerated dosing schedule for esmolol can then be tested for patient benefit in subsequent randomized controlled trials.

*Trial registration* ISRCTN, ISRCTN11038397, registered retrospectively 07/01/2021 https://www.isrctn.com/ISRCTN11038397

**Supplementary Information:**

The online version contains supplementary material available at 10.1007/s12028-023-01755-9.

## Introduction

Traumatic brain injury (TBI) is a global public health emergency. It is the leading cause of death in young adults, and it is a major cause of death and disability in all ages worldwide. The impact is felt by patients, families, and communities. In the United Kingdom, the annual cost is estimated at £15 billion, with more than 68,000 years of life lost [1, 2].

Neuroprotection is the preservation, salvage, or recovery of central nervous system function after acute insult. Better neuroprotective strategies would reduce death and disability after TBI. The current management strategies are conservative, relying on provision of physiological stability and timely management of complications (e.g., seizures, intracranial hypertension). There is no proven therapy that provides additional benefit [3].

There are plausible reasons to consider beta-blockers as potential neuroprotective drugs after TBI. Plasma catecholamine levels correlate with inflammation, endothelial injury, and poor outcome after TBI [4–6]. In animals, administering beta-blockers after TBI reduced cerebral edema and hypoxia (mice), increased perfusion (mice), and protected cerebral autoregulation (pigs) and it was associated with better recovery (mice) [7–9].

In patients a number of meta-analyses show that there is potential benefit for beta-blockade in TBI to reduce mortality and improve functional outcomes [10–14]. However, the studies are heterogenous and largely observational. A few small, randomized trials are supportive but have limited external validity and/or have not been subject to peer review [15–18]. This means there is still uncertainty about the overall effectiveness of beta-blockade as well as the specifics of patient selection, drug, dose, route, and physiological goal.

Repurposing an established beta-blocker for neuroprotection could significantly improve individuals’ health outcomes, reduce the impact on families and communities, and save resources for health systems and societies at a low cost, potentially representing excellent value for money. There is, however, a risk with the use of antihypertensive drugs early after severe TBI; compromising blood pressure maintenance in the group of patients with TBI, for whom hypotension has been called “the single most important secondary insult,” could lead to worse outcomes [19].

With this risk–benefit balance in mind, we designed the Early Beta-Blockade in adults after severe Traumatic Brain Injury (EBB-TBI) program, of which this is the first of three planned studies. The overarching hypothesis of the EBB-TBI program of research is that beta-1 adrenoceptor blockade after severe TBI in adults reduces morbidity and mortality by reducing secondary brain injury driven by the hyperadrenergic state. Here, we describe the protocol for the first study. We use methodology common to early phase drug studies to test escalating starting doses for esmolol infusion in small cohorts of patients with an end point of heart rate control without compromise of cerebral perfusion pressure (CPP). The aim is to optimize the intervention with esmolol so it can then be tested in subsequent trials of efficacy and effectiveness.

### Rationale for Choice of Esmolol and Dosing Regimen

There are several characteristics of esmolol that are well suited to use for beta-blockade in the early phase of critical illness. First and foremost, the rapid onset (time to steady state of action of 5 min), facilitates titration to the desired clinical end points. This, coupled with the short elimination half-life of 9 min, means rapid offset of effect if reduced or stopped for adverse events. The drug has few interactions with other medications beyond the general class effects of beta-1 selective-blockers. It is metabolized to inactive compounds, and this is independent of organ function. The intravenous route of administration bypasses the gut and guarantees drug delivery in a population where gastric emptying is frequently delayed. Finally, it is inexpensive, in common use in intensive care units (ICUs), and straightforward in its preparation and administration (e.g., it is stable at room temperature for up to 24 h, protection from light is not required, and it can be administered via peripheral venous access) [20, 21].

Although the primary reason for choosing esmolol is practical, there are also theoretical arguments. A beta-1 selective blocker (metoprolol) is used in the Lund approach to the management of TBI because it does not cause cerebral vasodilatation or alter cerebral blood flow after severe TBI [22, 23]. Esmolol itself has been shown to have no adverse effect on cerebral blood flow in volunteers or in patients receiving electroconvulsive therapy [24, 25]. In the setting of anesthesia, with the same drugs commonly used for sedation after TBI, esmolol provides additional cortical suppression that could contribute to neuroprotection [26]. In animals, esmolol is neuroprotective after brain or spinal cord ischemia [27, 28]. Finally, there is evidence that esmolol reduces the inflammatory response after surgical trauma [29].

The initial dosage selected for testing in the first cohorts in the EBB-TBI study (esmolol infusion started at 5 µg per kilogram per minute [mcg.kg.min^-1^]) is based on a regimen shown to be tolerated by a population with severe septic shock who are, like many patients after severe TBI, mechanically ventilated and receiving vasopressors [30]. The esmolol was then titrated against heart rate using dosage increments of 2.5 mcg.kg.min^-1^ every 30 min. The infusion starting rate for subsequent cohorts was determined by using the so-called modified Fibonacci sequence (commonly used in oncology dose-finding studies [31, 32]) using the initial EBB-TBI cohort dosage as the start of the sequence.

Dosages as low as 15 mcg.kg.min^−1^ are anti-inflammatory after surgical trauma in humans [29]. The effective concentration (EC50) for reduction in heart rate during exercise in humans is 113 mcg.kg.min^−1^ [33]. Side effects, mainly hypotension, are more common with dosages exceeding 150 min^−1^ [20, 33]. In rats a dosage of 20 mcg.kg.min^−1^ is protective against cerebral ischemia, and 16 mcg.kg.min^−1^ is anti-inflammatory in sepsis [34, 35]. Although the minimum dosage required for neuroprotection in humans is not known, this supports the concept that a dosage of esmolol resulting in heart rate reduction is also potentially effective in reducing brain injury and that neuroprotection might be possible while avoiding serious side effects.

Rather than a fixed dosage, the infusion will be titrated to heart rate, a convenient clinical biomarker for sympathetic nervous system activity and catecholamine drive, allowing for personalization of dosage. We will aim for a 15% reduction from preenrollment baseline. Titration of medication to achieve physiological goals is routine in intensive care practice. Use of heart rate avoids any conflict with accepted physiological goals like blood pressure set in clinical guidelines (such as those from the Brain Trauma Foundation [19]).

In healthy volunteers, heart rate reduction of 15% induced by esmolol did not alter cerebral blood flow [24]. After ST elevation myocardial infarction (STEMI), a 14% reduction in heart rate with esmolol did not increase the incidence of cardiogenic shock or atrioventricular block while limiting the peak cardiac troponin T release [36]. A 20% reduction in heart rate in septic mechanically ventilated patients did not alter oxygen utilization or hepatic or leg blood flow [37]. Evidence relating to the ideal target heart rate in severe TBI is conflicting [38, 39].

We aim to administer esmolol as soon as practicable after injury to achieve early blockade of the hyperadrenergic surge accompanying the head trauma and interruption of self-perpetuating pathophysiological cascades before secondary injury is established [40]. Observational data show catecholamine related pathophysiology at the time of hospital admission for TBI [5]. The duration of the infusion is limited to 4 days from enrollment. This translates into an intervention period that spans the time of initial development of cerebral edema after injury. This regimen is therefore based on a combination of clinical judgment of the time of greatest sympathetic activation with knowledge of the typical course of intracranial hypertension [41, 42]. It allows the primary research question for this study to be answered at the time of greatest potential hemodynamic instability without unnecessarily prolonged exposure to a drug that may not ultimately provide benefit. After this period, the continued use of esmolol or any other selective or nonselective beta-blocker is at the discretion of the treating clinician. To the best of our knowledge, there is no literature examining the optimal timing of administration of beta-blockers after traumatic, ischemic, or septic insult.

### Rationale for Use of Continual Reassessment Method

Dose-finding studies are used in early-phase research to estimate the dose-toxicity profile of drugs and to select the right dose for subsequent trials. Rule-based study designs rely on predefined rules to determine future dosage decisions based on observed toxicity at current doses. In contrast, model-based designs use statistical models to guide these decisions based on a target level of toxicity that combines judgments of the potential benefit of drug administration and severity of harm arising from toxicity.

The continual reassessment method (CRM) is a parametric model-based study design. Several advantages are reported for model-based designs like the CRM over traditional rule-based designs, including flexibility and increased precision. This aids an efficient study design that will minimize the number of patients required to determine the maximum tolerated dose and maximizes safety by exposing the fewest patients possible to either undertreatment or overtreatment and by rapid dose titration [43–46].

Additional safety measures are possible with the CRM without compromising the study performance [47]. Given the lack of data on early esmolol dosing in TBI, the uncertain magnitude of any benefit, and the potential for severe harm with toxicity (i.e., hypotension), this is important for this study. A run-in cohort of three patients, increased number of cohorts, and avoidance of skipping untried doses have been incorporated in this protocol.

### Study Outline

This is a prospective dose-finding study of esmolol aiming to attenuate the sympathetic surge associated with severe TBI in adults. The setting is the 48-bed mixed ICU of Southmead Hospital, Bristol, a 996-bed teaching hospital in the southwest of England, and a major trauma center serving an adult population of approximately 2.3 million. On average, the ICU admits more than 130 patients with severe TBI annually.

The primary objective is to define a treatment dosage escalation schedule for esmolol for use in adults early (< 24 h) after severe TBI that balances the potential benefit of early exposure to beta-blockade with the ability to maintain adequate CPP. A reduction of heart rate of at least 15% from preinfusion baseline will be used as an indicator of clinically significant beta-blockade.

A group sequential adaptive model-based design (the CRM) will be used to determine the maximum tolerated dosage schedule for esmolol, defined as the highest dosing regimen associated with an acceptable level of toxicity. For the purpose of this study, this is taken to be a probability of dose-limiting toxicity of 10%. Dose-limiting toxicity is defined as failure to maintain CPP above the minimum recommended by Brain Trauma Foundation guidelines (60 mm Hg), despite standard interventions including vasopressors and the protocolized deescalation of the esmolol infusion such that withdrawal of esmolol is required, or the occurrence of a serious adverse event mandating withdrawal of the esmolol infusion.

Secondary objectives are to identify the effects of esmolol on organ function and to record clinical outcomes including mortality and function at 6 months. Exploratory objectives are to monitor the effects on biomarkers of sympathetic nervous system activity and to gather data to inform the design of a randomized controlled trial to establish feasibility and efficacy.

### Study Flow Chart

**Fig. 1:**
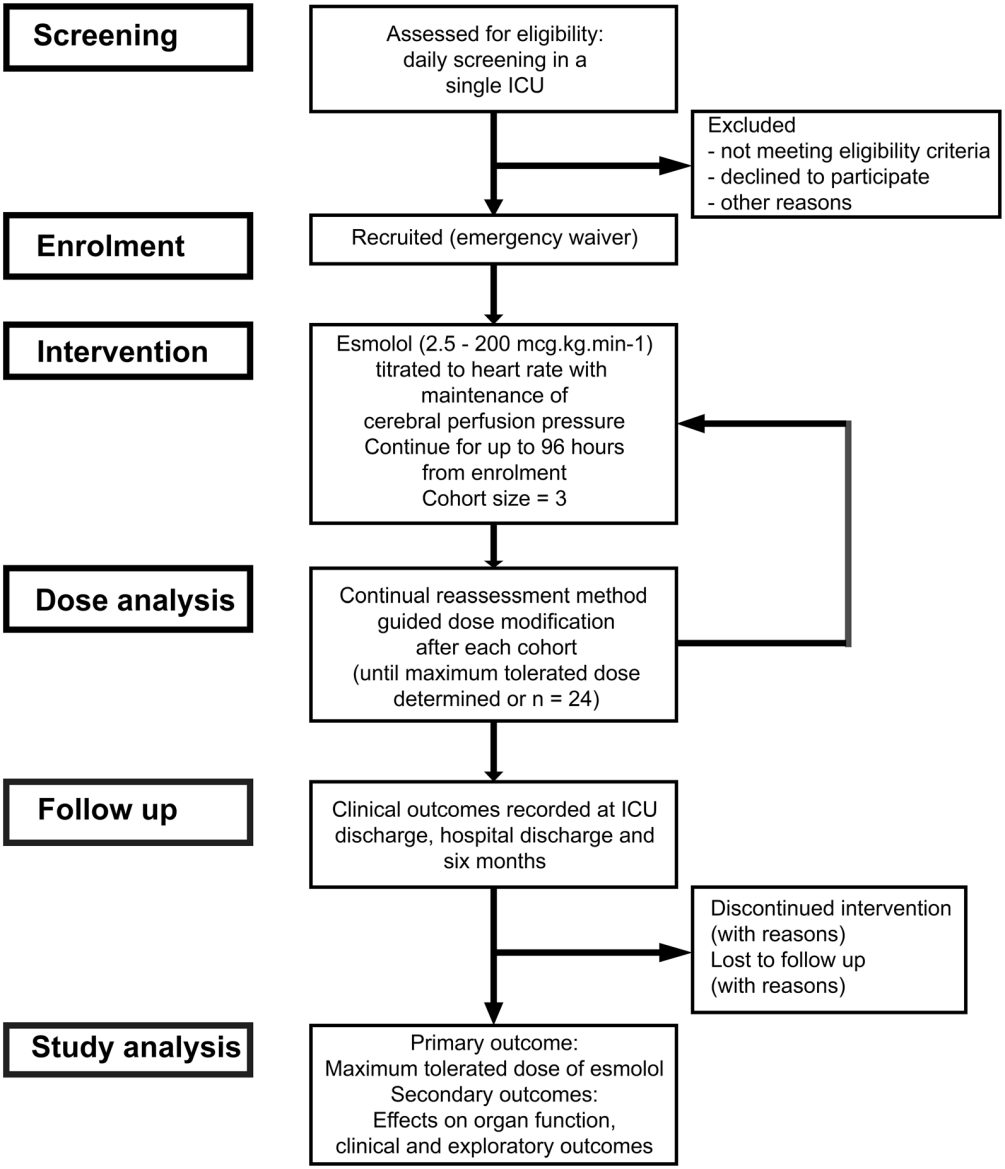
Flow of patients through the study. ICU, intensive care unit

Flow through the study is shown in Fig. 1.

### Eligibility

Participants must meet all inclusion criteria and none of the exclusion criteria (shown in Table 1) and start the esmolol infusion within 2 h of confirmation of eligibility. The baseline heart rate must be > 60 beats per minute for more than 15 min for the infusion to start.

**Table 1:** Eligibility criteria

Inclusion criteria (all to be met)	Exclusion criteria (none to be met)
1. Aged 18 years or more	1. Life or limb threatening extracranial injury (in the opinion of the treating intensivist)
2. Severe traumatic brain injury (Glasgow Coma Score of 8 or less after resuscitation or prior to intubation)	2. Perceived devastating brain injury admitted for the purposes of prognostication or organ donation
3. Within 24 h of injury	3. Participation in another clinical trial of investigational medicinal product within preceding 30 days
4. Intracranial pressure monitoring in situ	4. Pregnancy
	5. Breast feeding
	6. Hypersensitivity to beta-blockers
	7. Cardiogenic shock
	8. Decompensated heart failure (NYHA class 4)
	9. Untreated sick sinus syndrome or AV nodal conduction disorders including second-degree or third-degree heart block)
	10. Untreated phaeochromocytoma
	11. Acute severe bronchospasm
	12. Severe pulmonary hypertension (mean PA pressure > 55 mm Hg)
	13. Prinzmetal’s angina
	14. Severe metabolic acidosis (pH < 7.1)
	15. Use of verapamil within the preceding 48 h

AV, atrioventricular, PA, pulmonary artery, NYHA, New York Heart Association

These criteria exclude those at greatest risk of harm from beta-blockade, target early enrollment for greatest potential benefit and remain broad to capture a representative patient population.

### Consent

Potentially eligible participants, because of their severe TBI, will lack the capacity to provide consent for this study. The need for early intervention to maximize potential benefit, and the uncertainty of time and extent of recovery, means it is not practicable to wait for capacity to return. The time critical nature of the intervention and the potential for significant additional distress in the emergency situation precludes seeking prior personal legal representative opinion. As such, an emergency waiver of consent model will be used, with informed consent sought once patients regain capacity, as laid out in UK legislation (The Medicines for Human Use [Clinical Trials] Amendment [No.2] Regulations 2006).

Although any participant (or their legal representative) may withdraw their consent at any time, given the adaptive nature of the study with frequent analysis of patient data and the dependence of later study drug dosing on prior patient response, it is not feasible to withdraw all data unreservedly. Participant data that has been used in study drug dose calculation will not be withdrawn.

### Study Intervention

Open-label esmolol is given as a continuous intravenous infusion at a dosage of 2.5–200 mcg.kg.min^−1^ titrated using a predefined dosage escalation schedule to achieve a heart rate reduction of ≥ 15% from baseline with CPP maintained above 60 mm Hg. The target heart rate will be set as the rolling mean over the preceding 5 min to avoid overshoots. Baseline is defined as the mean heart rate in the 4 h preceding confirmation of eligibility. The minimum permitted target heart rate will be 60 beats per minute, even if this is less than 15% reduction from baseline; the maximum will be 100 beats per minute, even if this is more than a 15% reduction from baseline. Actual body weight at the time of enrollment in the study (estimated or known) will be used for dosage calculations.

A starting dosage is defined for each cohort, with dosage increments for that cohort being 50% of the starting dosage. The dosage is reviewed and adjusted every 30 min as required to achieve the target heart rate (± 5 beats per minute). Titration to achieve heart rate control for short lived stimulating procedures (e.g., tracheal suction, positioning, portable chest X-ray) is not required. The infusion should be continued during procedures including surgery or within hospital transfers for imaging.

When the infusion is restarted after temporary suspension (e.g., for bradycardia or if heart rate maintained in target range without need for infusion), the starting dosage for that level will be used with increments every 30 min as required. The infusion should not be restarted after a temporary suspension until the heart rate exceeds the minimum target rate (or 60 beats per minute) for more than 15 min.

Esmolol infusion continues according to study protocol until one of the following stopping rules is met:

- Ninety-six hours from start of infusion
- Heart rate target achieved without esmolol for > 12 h
- Dose-limiting toxicity
- Death or withdrawal of life-sustaining treatment
- Request of participant, legal representative, or treating clinician
- ICU discharge or transfer to nonparticipating ICU

The esmolol is weaned in steps of 5–10 mcg.kg.min^−1^ every hour from 96 h to avoid rebound tachycardia. In the event of dose-limiting toxicity, it may be reduced more quickly or stopped immediately. In the event of ICU discharge or transfer to a nonparticipating ICU, the infusion will be reduced at a rate calculated to ensure at least 2 h without esmolol infusion prior to discharge.

Bradycardia is defined for the purposes of this study as a heart rate under 50 beats per minute. The dosage of esmolol should be reduced in the appropriate increments for dosage level every 30 min until the bradycardia resolves.

Bradycardia with hemodynamic compromise is a heart rate under 50 beats per minute and a systolic blood pressure under 110 mm Hg (or 100 mm Hg for those aged 50–69 years). The dosage of esmolol should be reduced by twice the appropriate increment for dosage level every 30 min until bradycardia with hemodynamic compromise resolves.

When bradycardia is severe (defined as heart rate under 30 beats per minute), the esmolol infusion may be stopped temporarily until bradycardia resolves. Further intervention is at the discretion of the clinical team including intravenous antimuscarinic or chronotropic drugs and external or transvenous pacing.

Second-degree heart block without bradycardia or hemodynamic compromise should be managed by weaning esmolol as at the end of the intervention period. If hemodynamic compromise occurs, or in the event of third-degree heart block, management is the same as for severe bradycardia.

Hypotension should be managed according to usual clinical practice, taking into account cardiac status and CPP target. Fluid resuscitation and use of vasoactive agents including catecholamine and noncatecholamine vasopressors and inodilators are permitted. Esmolol infusion should be reduced in increments if these measures are insufficient to maintain CPP.

**Fig. 2:**
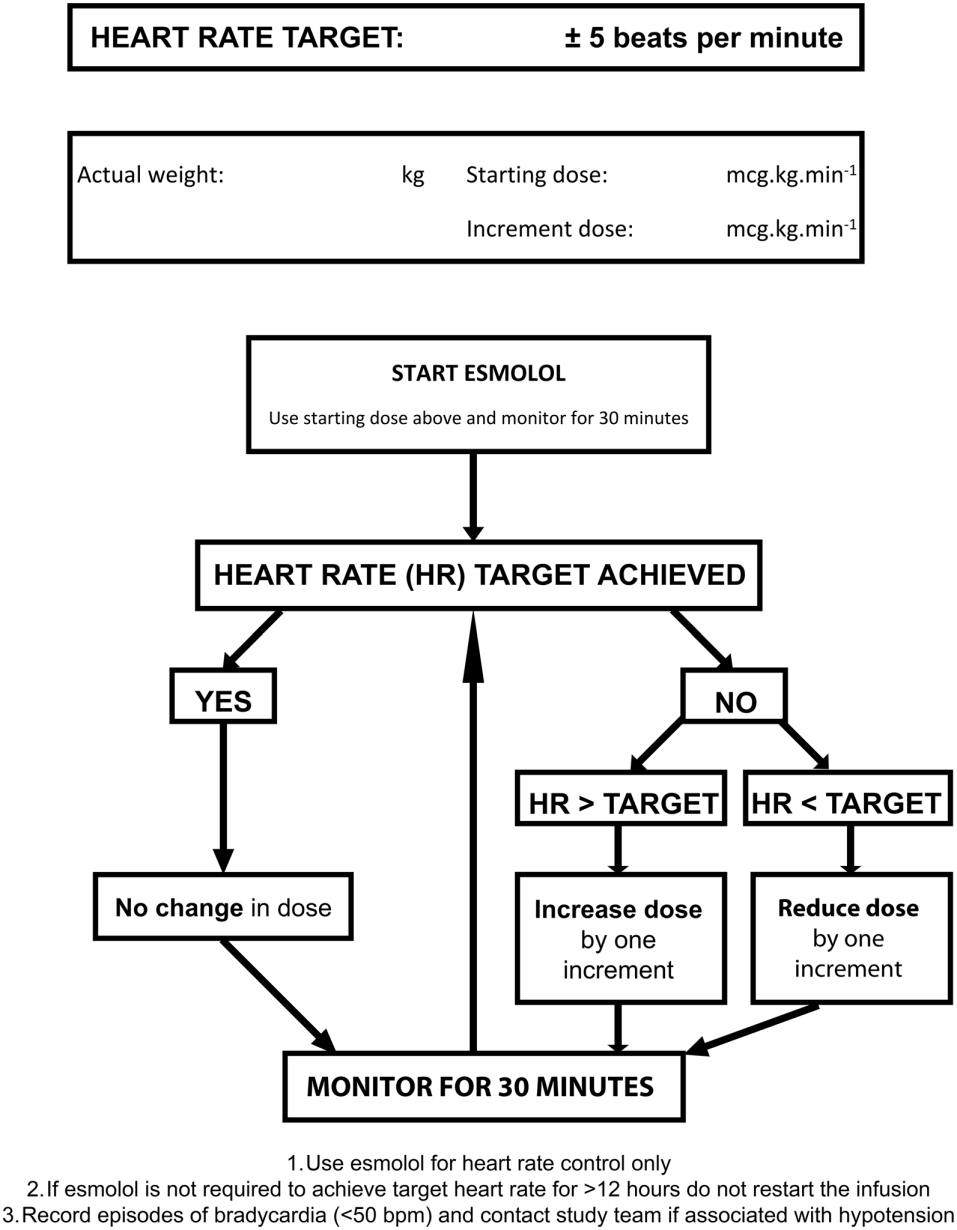
Flowchart for titration of esmolol dose. bpm, beats per minute, HR, heart rate

**Fig. 3:**
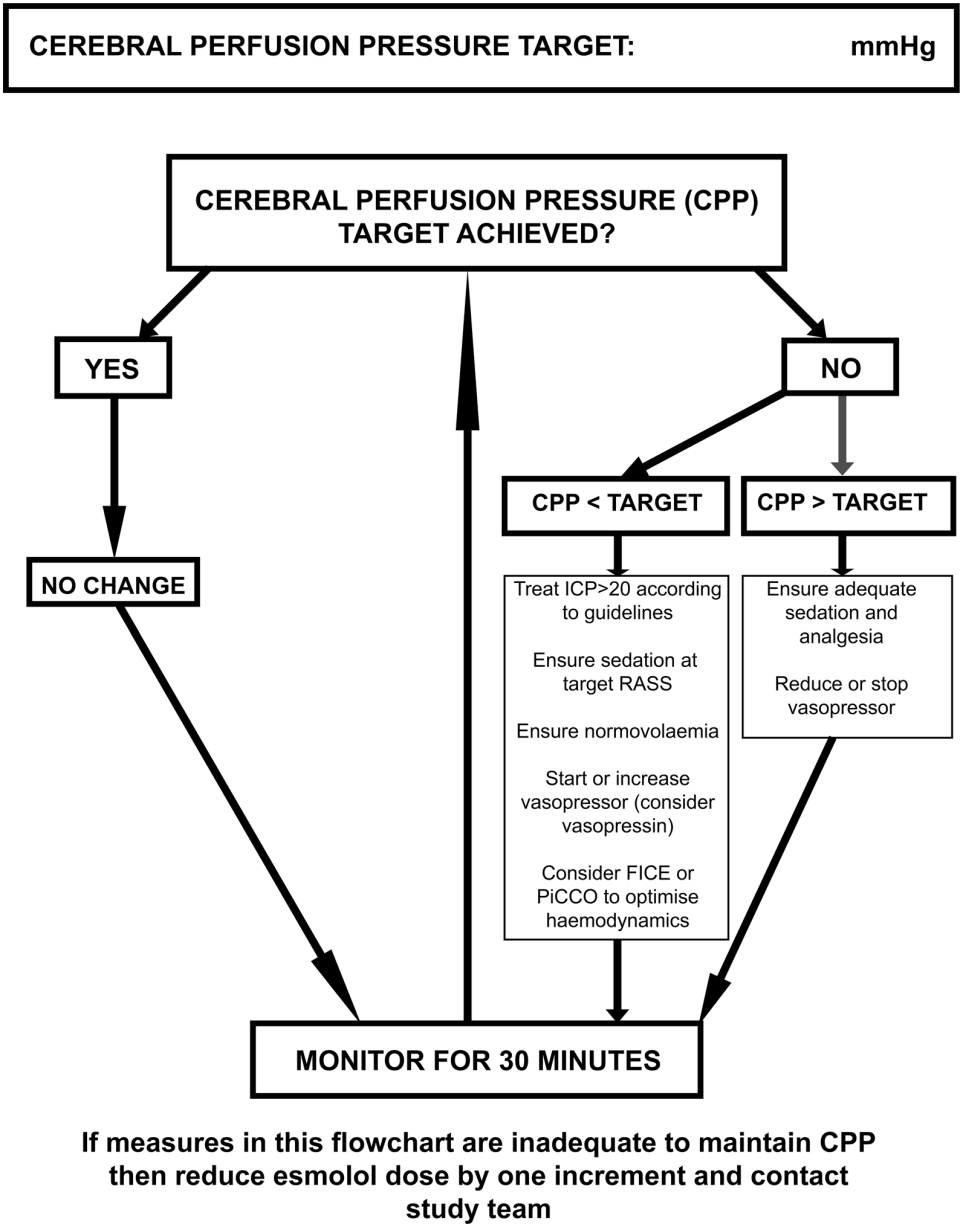
Flowchart for management of CPP. CPP, cerebral perfusion pressure, FICE, focused intensive care echocardiography, ICP, intracranial pressure, PiCCO, pulse index continuous cardiac output, RASS, Richmond Agitation Sedation Scale

Flow charts for the management of esmolol infusion are shown in Figs. 2 and 3.

### Concomitant Interventions

Enteral or parenteral use of selective or nonselective beta-adrenergic blockers is not permitted during the intervention phase (i.e., during esmolol infusion including weaning period). Given the short elimination half-life of esmolol, a 2-h gap from termination of infusion is considered sufficient. No other specifications on the use of concomitant interventions are made.

Standard TBI management at Southmead Hospital is based on Brain Trauma Foundation guidelines [19]. Arterial pressure transducers are zeroed at the level of the external auditory meatus. Multimodality neuromonitoring is not used.

### Baseline, Intervention, and Follow-Up Data

Baseline demographics collected will include age, gender, Glasgow Coma Score, time of injury and admission, Charlson comorbidity index, beta-blocker use at admission, intracranial and extracranial injury (abbreviated injury score and injury severity score), and the Helsinki computed tomography (CT) score. Intracranial pressure directed interventions include osmotic therapy, sedation and neuromuscular blockade, hyperventilation, therapeutic hypothermia (deliberate reduction in core temperature below 35 °C), radiological investigation of elevated intracranial pressure, cerebrospinal fluid (CSF) drainage, and craniotomy or craniectomy. The schedule of assessments is shown in full in Table S1 (Supplementary Material).

### Primary Outcome

The primary end point is a continual reassessment method-derived maximum tolerated dosage escalation schedule for esmolol that combines clinically significant reduction in heart rate (defined as ≥ 15% from baseline) with maintenance of CPP.

### Secondary Outcomes

Secondary outcomes in this study are the following:

1. Organ function
  Sequential organ failure assessment (excluding neurological assessment)
2. Clinical
  Mortality: ICU, acute hospital, and 6-months
  Length of stay: ICU and acute hospital
  Duration of mechanical ventilation
  Bloodstream infection in ICU
  Extended Glasgow Outcome Score (eGOS) at 6 months
  Quality of life (EQ-5D-5L) at 6 months

### Exploratory Outcomes

Biomarkers will include cardiac troponin T, coagulation screen, glucose, lactate, and heart rate. Blood will be stored for subsequent analysis of further endothelial and other biomarkers.

Estimates of efficacy and feasibility, other than the clinical outcomes listed above, will include the following:

1. Safety
  Incidence of bradycardia (heart rate < 50 beats per minute) with or without hemodynamic compromise requiring intervention other than reduction of esmolol dosage
  Incidence of second-degree or third-degree heart block with or without hemodynamic compromise requiring intervention other than reduction of esmolol dosage
  Incidence of clinically significant hypotension (systolic blood pressure < 100 mm Hg for patients aged 50–69 years, < 110 mm Hg for others) requiring intervention other than reduction of esmolol dosage
2. Efficacy
  Dose and duration of vasopressor during esmolol infusion
  Proportion of time during esmolol infusion with CPP in target range (60–70 mm Hg)
  Number of interventions per calendar day for intracranial pressure control during esmolol infusion (with daily and domain therapy intensity level scores)
3. Feasibility
  Rates of recruitment, consent after emergency waiver and loss to follow-up
  Noncompliance with study protocol
4. Acceptability

Additional funding will be sought for studies to support exploratory outcomes including biomarker analysis of stored blood and qualitative investigation of study acceptability and protocol delivery.

### Pharmacovigilance

Patients admitted to intensive care following severe TBI are critically ill and have a high baseline risk of complications of illness and of death. Medical occurrences that meet the definition of adverse events and adverse reactions may be expected features of critical illness requiring ICU care. All adverse events and adverse reactions will be considered in the context of the individual patient’s clinical condition and the natural history of severe TBI. Those who are considered by the chief investigator (or medically qualified designate) to be consistent with the patient’s critical illness do not require recording or reporting, unless the investigator considers they may relate to participation in the trial. All serious adverse events and serious adverse reactions both expected and unexpected will be recorded. For the purpose of this study, the Reference Safety Information is the Summary of Prescribing Characteristics for Brevibloc Premixed 10 mg/mL solution for infusion (Baxter Healthcare Ltd) [21].

Secondary and exploratory outcomes include assessment of organ function and significant hemodynamic side effects of esmolol infusion as a means of capturing additional safety information in the patient population. Protocol-based guidance is available for the management of specific adverse events (e.g., bradycardia with hemodynamic compromise). The primary responsibility for management of adverse events lies with the treating clinician.

### Statistical Analysis

Inability to maintain CPP in the presence of esmolol was chosen as the primary definition of toxicity based on the known importance of adequate blood pressure for prevention of secondary brain injury [19]. The maintenance of adequate CPP as a key goal of therapy is also familiar to clinicians practicing in the field of neurointensive care.

Only data from esmolol-treated patients will be used in statistical analysis. Esmolol-treated patients are those who meet all inclusion criteria and no exclusion criteria, receive any dosage of esmolol within 2 h of confirmation of eligibility and 24 h of injury, and do not withdraw consent for use of data prior to use of that data in CRM analysis.

A one-parameter logistic model, initialized with skeleton parameters as per Table 2, will be used for the CRM modeling, with estimated probabilities revised as data emerge. This likelihood modeling algorithm will identify a maximum tolerated dose escalation schedule, with a defined prior reasoned target toxicity level, or “acceptable” toxicity rate (*θ*), of 10% with an indifference level of 2 percentage points for decision making. The weighting afforded to the pretrial logistic model on estimated probabilities will be revised as trial data sequentially emerge.

**Table 2:** Predefined dose levels for esmolol infusion

Dose level	Starting dosage (mcg.kg.min^−1^)	Increment (mcg.kg.min^−1^)	Estimated prior probability of dose-limiting toxicity
1	5	2.5	0.01
2	10	5	0.04
3	16	8	0.07
4	25	12.5	0.10
5	35	17.5	0.15
6	46	23	0.20
7	62	31	0.25

Cohort size of three will be used. In the absence of dose-limiting toxicity, an increase in the dosage escalation schedule will be considered by the trial management group after the last patient in a cohort has completed the esmolol intervention period. Dosage escalation cannot be by more than one level and, as an additional safety measure, the dosage will not be escalated until the second cohort has completed intervention. Dosage deescalation will be considered after each dose-limiting toxicity and is unrestricted. Dosage level changes are subject to sponsor approval with oversight of dosage decision making the responsibility of the Steering Committee comprising independent expert and lay members. The Steering Committee is also responsible for monitoring study conduct and safety in lieu of formal Data Monitoring and Ethics Committee given the setting and design of the study.

The planned sample size of 24 esmolol-treated patients was determined in a pragmatic manner based on estimated recruitment rates following a test screening period in the ICU that were compatible with the timeline required by the funder and on the expected small information gain for additional patients over and above the target. This allows testing of up to seven dosage levels (Table 2).

In analysis of secondary and exploratory outcomes continuous variables will be summarized by descriptive statistics (mean and standard deviation, minimum, median, maximum, and interquartile range) and categorical data will be summarized in terms of frequency and percentage.

The Sequential Organ Failure Assessment will be reported as both daily total and by variable, excluding neurological assessment. Mortality outcomes will be analyzed using Kaplan–Meier survivorship. Length of stay and duration of mechanical ventilation will be analyzed using time to event Kaplan–Meier analyses. Both the number of patients with a bloodstream infection and the total number of bloodstream infections in ICU will be reported. The eGOS will be reported by category and dichotomized into favorable (eGOS 4–8) and unfavorable (eGOS 1–3) outcome, differentiating between patients who are independent at home or who are not. Quality of life (EQ-5D-5L) will be reported as the EQ-5D index and by each dimension.

Blood biomarkers (troponin, glucose, lactate, and International Normalized Ratio) will be reported for each day of the esmolol infusion. The incidence of safety outcomes is the number of events per calendar day of esmolol infusion (i.e., with at least 12 h of infusion) and will be reported as an overall incidence and by category. The dosage of vasopressor will be reported as noradrenaline equivalents in micrograms per kilogram per minute, with a correction factor of 10 used to convert from metaraminol.

The rate of recruitment reported as percent of screened population enrolled and as % of eligible population enrolled by month. Noncompliance with protocol, defined according to Sponsor’s Standard Operating Procedure as any breach of Good Clinical Practice or protocol, will be presented as total number of events and number of patients with episodes of noncompliance.

No subgroup or adjusted analyses are planned. There will be no imputation for missing data, which will be recorded as missing if queries are unable to recover the data. Some types of missing data represent study outcomes and will be reported as such.

Further quantitative analysis of relationships between outcomes or other study data will be undertaken with appropriate statistical methods. Qualitative analysis of acceptability will be undertaken using constant comparison methodology adapted from grounded theory with further details available via the Open Science Framework registration (https://osf.io/9ht4v). After publication, the data will be made available to other researchers on request if approved by the Trial Management Group and Sponsor.

### Funding, Sponsorship, and Ethical Review

The study is funded by the Research for Patient Benefit program of the National Institute for Health and Care Research (Award PB-PG-0418-20,029). The views expressed in this article are those of the authors and not necessarily those of the National Institute for Health and Care Research or the Department of Health and Social Care. The Sponsor is North Bristol NHS Trust. The study was approved by South Central—Hampshire A Research Ethics Committee (reference 20/SC/0219).

### Recruitment

The first participant enrollment occurred on 30th December, 2020, with the last participant final follow-up expected by 30th April, 2023.

### Study Registration

The study is registered with the International Standard Randomised Controlled Trial Number (ISRCTN) Registry (ISRCTN11038397).

## Discussion

There is a signal suggesting a significant benefit associated with beta-blockade after severe TBI seen in several meta-analyses, although the ideal drug and dosage schedule has not been determined. The EBB-TBI research program aims to define and test an intervention package based on esmolol. This first study aims to determine a maximum tolerated dosage of esmolol given that we are aiming to institute clinically significant beta-blockade at a time where there is a real risk of exacerbating secondary brain injury through hypotension.

Although some analyses favor the use of propranolol, a nonselective beta-antagonist, over other beta-blockers the data do not come from prospective randomized trials and it is not possible to eliminate potential confounders [48, 49]. Further, propranolol is compared against all other beta-blockers including other nonselective drugs as well as those with alpha-antagonist or class III antiarrhythmic actions (labetalol and sotalol respectively). As the authors acknowledge, these studies leave the question of the ideal drug open. We believe the practical advantages of esmolol together with the potential theoretical benefits make it an ideal drug for use in the early period after severe TBI [20–29, 34, 35].

Use of the CRM, an adaptive model-based design for dose-finding studies, is intended to deliver a more efficient and safer study. In particular, in a patient population where the margin for error is narrow and the potential harm permanent and profound, and where the intervention challenges long held beliefs about clinical practice, minimizing the exposure of patients to potential toxicity is important. Our assumptions are based on a maximum dosage with only 10% acceptable probability of toxicity, against the 33% standard used in many phase-I trials [44, 50]. We believe that this approach is reasonable in this patient population given the balance of estimates of a dosage that might provide benefit against one which leads to the known harm of hypotension [20, 29, 33–35].

The strengths of this study include recruitment of patients not enrolled in other studies of beta-blockade after severe TBI (those with extracranial injuries, previous beta-blocker use or on vasopressors), early administration of intervention and an adaptive design. The intervention itself is simple and uses a drug that has rapid offset in the event of side effect or toxicity.

There are several limitations. Practice in a single center cannot be assumed to generalize more widely. Esmolol may not be available in all countries. We have used a simple surrogate of sympathetic activity and have not controlled for influences such as the adequacy of sedation or fluid resuscitation. Our sample size was determined pragmatically rather than formally estimated. The clinical benefit of esmolol cannot be determined as there is no control group and some relevant outcomes will not be collected. Similarly, we have not attempted to investigate mechanisms of effect or whether subgroups of the heterogenous TBI population might derive particular benefit or harm from the intervention, for example based on admission troponin [51]. We plan to address these limitations in subsequent studies in the program focused on efficacy and effectiveness.

## Conclusions

Here, we present the protocol for a dose-finding study of esmolol for early beta-blockade after severe TBI using the CRM. Both the drug and study design are novel in this setting. This study will determine a dosage schedule for esmolol that can be tested for benefit in adults with severe TBI.

### Supplementary Information

Below is the link to the electronic supplementary material.

Supplementary file1 (DOCX 18 KB)
